# Inhibition of Cdc42 activity extends lifespan and decreases circulating inflammatory cytokines in aged female C57BL/6 mice

**DOI:** 10.1111/acel.13208

**Published:** 2020-08-04

**Authors:** Maria Carolina Florian, Hanna Leins, Michael Gobs, Yang Han, Gina Marka, Karin Soller, Angelika Vollmer, Vadim Sakk, Kalpana J. Nattamai, Ahmad Rayes, Xueheng Zhao, Kenneth Setchell, Medhanie Mulaw, Wolfgang Wagner, Yi Zheng, Hartmut Geiger

**Affiliations:** ^1^ Program of Regenerative Medicine IDIBELL Barcelona Spain; ^2^ Institute of Molecular Medicine and Stem Cell Aging Ulm University Ulm Germany; ^3^ Helmholtz‐Institute for Biomedical Engineering Stem Cell Biology and Cellular Engineering RWTH Aachen University Medical School Aachen Germany; ^4^ Division of Experimental Hematology and Cancer Biology Cincinnati Children's Hospital Medical Center University of Cincinnati Cincinnati Ohio USA; ^5^ Division of Pathology Cincinnati Children's Hospital Medical Center University of Cincinnati Cincinnati Ohio USA; ^6^ Institute of Experimental Cancer Research Medical Faculty University of Ulm Ulm Germany

**Keywords:** aging, Cdc42, epigenetic clock, inflammaging, interferon gamma, lifespan

## Abstract

Cdc42 is a small RhoGTPase regulating multiple functions in eukaryotic cells. The activity of Cdc42 is significantly elevated in several tissues of aged mice, while the Cdc42 gain‐of‐activity mouse model presents with a premature aging‐like phenotype and with decreased lifespan. These data suggest a causal connection between elevated activity of Cdc42, aging, and reduced lifespan. Here, we demonstrate that systemic treatment of aged (75‐week‐old) female C57BL/6 mice with a Cdc42 activity‐specific inhibitor (CASIN) for 4 consecutive days significantly extends average and maximum lifespan. Moreover, aged CASIN‐treated animals displayed a youthful level of the aging‐associated cytokines IL‐1β, IL‐1α, and INFγ in serum and a significantly younger epigenetic clock as based on DNA methylation levels in blood cells. Overall, our data show that systemic administration of CASIN to reduce Cdc42 activity in aged mice extends murine lifespan.

## Graphical abstract

The activity of the small RhoGTPase Cdc42 is significantly elevated in blood of elderly humans and in several tissues of aged mice. Cdc42 constitutive activation induces premature aging and decreases murine lifespan. Here, we show that systemic treatment of aged (75‐week‐old) female C57BL/6 mice with a Cdc42 activity‐specific inhibitor (CASIN) for 4 consecutive days significantly extends average and maximum lifespan, reduces levels of IL‐1b, IL‐1a, and INFg aging‐associated cytokines in serum, and rejuvenates the biological epigenetic clock as based on DNA methylation levels in blood cells.

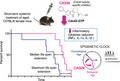

## 1 INTRODUCTION

Cdc42 is a small RhoGTPase involved in multiple and diverse functions of eukaryotic cells, including actin cytoskeleton reorganization, cell polarity, and cell growth (Cerione, 2004; Etienne‐Manneville & Hall, 2002). Cdc42 cycles between an inactive, GDP‐bound state, and an active, GTP‐bound state. The activity of Cdc42 and thus the level of Cdc42‐GTP are significantly elevated in blood of elderly humans (Florian et al., 2017) and in several tissues of aged C57BL/6 animals, including heart, brain, lung, liver, bone marrow, spleen, and kidney (Wang, Yang, Debidda, Witte, & Zheng, 2007). The cycling between the GDP‐bound form and the GTP‐bound form is tightly controlled by a number of different regulatory proteins. Cdc42 GTPase‐activating protein (Cdc42GAP; also known as p50RhoGAP or ARHGAP1) is a ubiquitously expressed negative regulator of Cdc42 that catalyzes hydrolysis of GTP bound to Cdc42 (Moon & Zheng, 2003). Genetic deletion of Cdc42GAP in mice (Cdc42GAP knock out) results in an elevated level of Cdc42‐GTP in every tissue. This constitutive increase of Cdc42 activity in young mice leads to a premature aging‐like phenotype that affects several tissues and results in a decrease in lifespan (Wang et al., 2007). All together, these data raise the possibility that the increase in Cdc42 activity might be causatively involved in regulating lifespan (Wang et al., 2007).

We recently identified a specific small‐molecule inhibitor of Cdc42 activity termed CASIN (Cdc42 activity‐specific inhibitor) (Liu et al., 2019). Modulation of Cdc42 activity by CASIN has been already applied to study the biological and pathological roles of Cdc42 in murine and human cells ex vivo (Florian et al., 2013, 2018; Grigoryan et al., 2018; Leins et al., 2018; Umbayev et al., 2018; Yang et al., 2019) as well as in tissues and mice in vivo (Du et al., 2018; Liu et al., 2019; Yang et al., 2019). Administration of CASIN in vivo did not show signs of toxicity (Du et al., 2018). Previously, we reported that a brief (16 h) ex vivo exposure of aged hematopoietic stem cells (HSCs) to CASIN that reduced the activity of Cdc42 in aged cells to the level found in young cells resulted in long‐lasting youthful function of HSCs in vivo, likely due to epigenetic remodeling of aged cells upon modulation of Cdc42 activity (Florian et al., 2012, 2018; Grigoryan et al., 2018). Consequently, we hypothesized that maybe a short‐term systemic reduction of Cdc42 activity in aged animals in vivo might be also beneficial for lifespan, as an elevated activity of Cdc42 upon aging is causatively linked to a shorter lifespan in mice.

**FIGURE 1:**
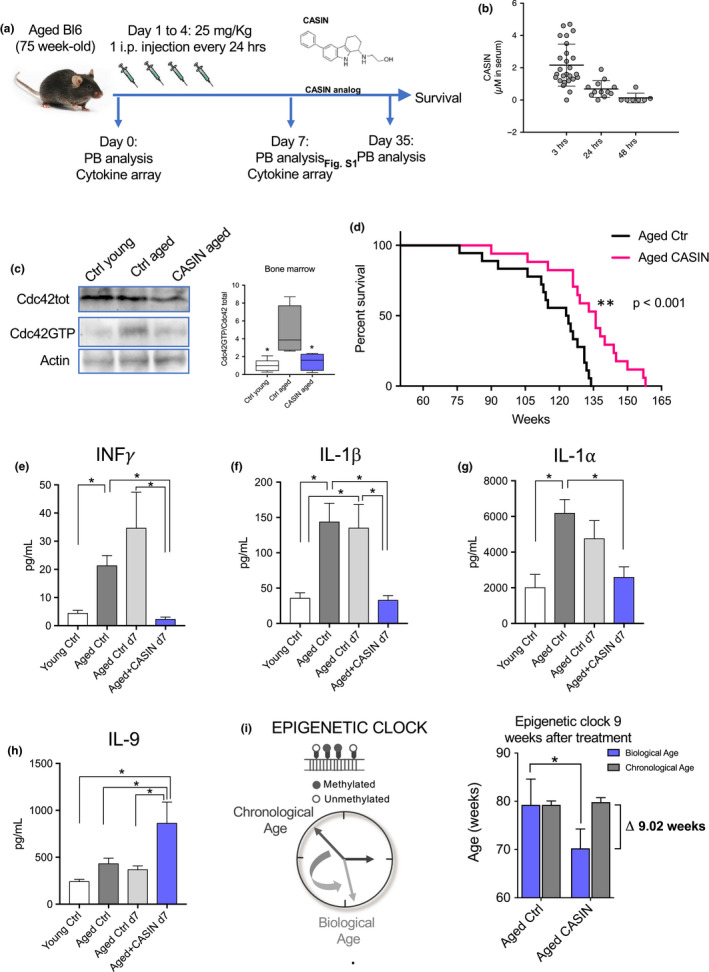
(a) Cartoon scheme depicting the experimental layout of the lifespan study. (b) Quantification by mass spectrometry of CASIN concentration in serum over time in 75‐week‐old C57BL/6 mice injected with a i.p. dose of 25 mg/kg of CASIN for 4 consecutive days every 24 h. The blood was harvested 3, 24, and 48 h after the last injection on day 4. *n* = 26 for the 3‐h time point, 12 for the 24‐h time point, and 7 for the 48‐h time point. (c) Representative image and quantification of Western blot/pull‐down of Cdc42 total and active (Cdc42GTP) in bone marrow cells from young (10‐week‐old) and aged (75‐week‐old) control and CASIN‐treated C57BL/6 mice. *n* = 4 mice per group; *p < 0.05 vs aged control according to one‐way ANOVA and Tukey's multiple comparisons test. (d) Survival curve for aged control (black) and CASIN (red)‐treated mice. Treatment was done according to the cartoon scheme depicted in panel a. *n* = 18 for control and 17 for CASIN; *p* < 0.0004 according to Mantel–Cox test and *p* < 0.0032 according to Gehan–Breslow–Wilcoxon test. Median survival for control 123.5 weeks and 136 weeks for CASIN. (e–h) Results from the cytokine array. Serum was collected from the same mice at day 0 (aged control) and day 7 (aged control and aged CASIN) according to the cartoon scheme in panel a. 7 young (10‐week‐old) C57BL/6 female mice were bled together with the old mice at day 0, and the serum was used for the cytokine array young control sample. The number of experimental mice included was *n* = 7 for young control, 27 for aged control day 0, 9 for aged control day 7, and 10 for aged CASIN day 7. The serum samples were loaded simultaneously for all cytokines probed and all experimental arms. Some samples did not generate a signal above the background due to technical reasons and were excluded from the statistical analysis. Bars represent mean ± *SEM*. *p < 0.05; **p < 0.01 according to one‐way ANOVA and Tukey's multiple comparisons test or Kruskal–Wallis test followed by Dunn's multiple comparison test for non‐Gaussian distributed data. (i) Biological age prediction as based on DNA methylation profile of blood cells from aged control and aged CASIN‐treated mice 8–9 weeks after treatment. The experiment was repeated twice with a cohort of 5–6 animals per group (*n* = 12 for control and 11 for CASIN). Bars represent mean ± *SEM*. *p < 0.05 according to unpaired *t* test analysis

To determine whether a short‐term systemic CASIN treatment of aged animals might indeed influence lifespan, we administered CASIN i.p. every 24 h for 4 consecutive days to 75‐week‐old female C57BL/6 mice (Figure 1a). CASIN levels in serum were monitored via liquid chromatography–mass spectrometry (Figure S1a,b). 50 mg/kg as single dose resulted initially in a concentration of about 10 µM in serum (data not shown). Our previously published data demonstrated a functional rejuvenation of aged BM cells at a concentration of 5 µM of CASIN (Florian et al., 2012, 2018; Grigoryan et al., 2018; Liu et al., 2019). Therefore, for the lifespan study we settled on a dosage of 25 mg/kg, and mass spectrometry data from serum of old mice showed levels of CASIN 3 h after injection in the expected µM range (Figure 1b). 4 days of consecutive injections did not induce acute toxicity, and as well, none of the treated mice died within 4 weeks after CASIN injections, rendering also chronic toxicity issues unlikely. Quantification of Cdc42 activity 24 h after the last injection on day 5 demonstrated a reduction of Cdc42‐GTP in aged bone marrow cells to the level seen in young (Figure 1c), confirming that CASIN is indeed reducing Cdc42 activity after a systemic in vivo treatment. Notably, aged mice treated with CASIN for only 4 consecutive days showed extension of their average and also maximum lifespan (Figure 1d). A long‐term influence of a short‐term treatment is consistent with our previous findings that transient changes in Cdc42 activity can modify epigenetic signatures in cells, conferring long‐term functional changes (Florian et al., 2012, 2018; Grigoryan et al., 2018; Leins et al., 2018).

Interestingly, weight (Figure S1c), white blood cell count (WBC) (Figure S1d), red blood cell count (RBC) (Figure S1e), and the frequency and count of myeloid and lymphoid cells in PB did not change in response to CASIN treatment, both short term (day 7 after beginning of the treatment) and long term (day 35 after beginning of the treatment) (Figure S1f–k). Aging in mice and humans has been associated with a general alteration in the concentration of inflammatory cytokines, termed inflammaging (Franceschi et al., 2000; Franceschi & Campisi, 2014; Minciullo et al., 2016) or, more recently, aging‐associated immune remodeling (AAIR) (Leins et al., 2018). We performed cytokine array analyses to investigate the extent to which aging‐associated inflammatory cytokines in serum of aged mice were affected by CASIN treatment. Data showed a marked increase in the concentrations of INFγ, IL‐1β, and IL‐1α on aging and the concentrations for these cytokines were similar to concentrations in young animals upon CASIN treatment of aged mice (Figure 1e–g). It is thus a possibility that a reduction in the concentrations of these cytokines upon CASIN treatment might contribute to the increase in lifespan observed in these animals. Interestingly, IL‐9 was the only cytokine analyzed that increased in serum concentration after CASIN treatment (Figure 1h). The concentrations of IL‐6, IL‐2, GM‐CSF, LIF, IL‐4, and IL‐7 showed a not significant trend to increase in aged mice and some (GM‐CSF, IL‐2) also a trend to be reduced by CASIN, while for a large number of cytokines their level in serum was not altered at all either by aging or CASIN treatment (Figure S2).

Previously, the methylation status of CpG sites within the genes *Prima1*,* Hsf4*,* and Kcns1* was shown to qualify as likely predictor of biological age of C57BL/6 mice (Han et al., 2018). Therefore, DNA methylation profiling of these CpGs might serve as biological epigenetic clock (Bell et al., 2019). Applying this C57BL/6‐trained DNA methylation marker panel to blood cells from aged animals treated with CASIN 9 weeks after treatment, we observed that epigenetic age predictions did not correlate anymore to the chronological age as in aged control animals, but resulted in a biological age prediction that was on average 9 weeks younger than their chronological age (Figure 1i). These data imply that epigenetic changes underlie the extended longevity of aged CASIN‐treated mice, while reinforcing the necessity to mechanistically validate tissues, cells, and biological pathways involved in the extension of longevity.

Interestingly, a recent analysis of changes in the epigenome and transcriptome with aging across different tissues identified that the up‐regulation of the inflammatory response and gamma interferon pathways correlated best with aging both across tissues and species (Benayoun et al., 2019). Many age‐related diseases, such as obesity, metabolic syndrome, diabetes, cardiovascular diseases, cancer, depression, and Alzheimer's disease, share an inflammatory pathogenesis; moreover, the activation of inflammatory pathways appears to be involved in the pathophysiology of sarcopenia and frailty. Elevated concentrations of interferon γ, as detected in our study, might thus serve as a conserved hallmark of aging and might be functionally linked to lifespan. Similarly, both interleukin 1α and interleukin 1β have been associated with many aging‐associated phenotypes and diseases (Mantovani, Dinarello, Molgora, & Garlanda, 2019). As for IL‐9, which showed an increase in the concentration in serum after CASIN treatment, it is a pleiotropic cytokine mainly produced by T helper 9 (Th9) cells and exerts documented effects on lymphocytes, mast cells, and resident lung cells (Goswami & Kaplan, 2011). Our data further support a role for these cytokines in the regulation of lifespan. Moreover, our data reveal that the methylation status of CpG sites within the genes *Prima1*,* Hsf4*, and *Kcns1* in blood cells strongly correlates with biological age and might thus serve as a validated biomarker of aging in C57BL/6 mice.

Multiple studies in organisms ranging from flies and worms to humans have demonstrated that genetics plays an important role in determining lifespan (Kuningas et al., 2008), while there are robust and reproducible approaches to extend lifespan and healthspan by non‐genetic means like dietary restriction (Longo et al., 2015; Mercken et al., 2013; Mitchell et al., 2016) or pharmacological compounds (https://www.nia.nih.gov/research/dab/interventions‐testing‐program‐itp) (Campisi et al., 2019; Palliyaguru, Moats, Di Germanio, Bernier, & de Cabo, 2019). Cdc42 has been implied in the regulation in multiple organisms, ranging from yeast and *Caenorhabditis elegans* to humans (Florian et al., 2017; Kerber, O’Brien, & Cawthon, 2009; Meitinger et al., 2014; Witten & Bonchev, 2007). We demonstrate here that a reduction of Cdc42 activity in aged mice indeed extends lifespan. Cdc42 might thus be similar to other phylogenetically conserved genes and pathways (Fontana, Partridge, & Longo, 2010; Kennedy & Lamming, 2016; Kuningas et al., 2008), a critical target in the context of longevity. Interestingly, to the best of our knowledge, inhibition of Cdc42 activity by CASIN and inhibition of mTOR by rapamycin are the only pharmacological treatments reported so far that have significant effects on murine lifespan when given already quite late in life (75 weeks of age or more) and only temporary (only 4 days for CASIN; 90 days for rapamycin) (Bitto et al., 2016). This argues for a mechanism of action of CASIN that is distinct from the other pharmacological interventions for lifespan, which usually are either given earlier in life and/or then provided continuously for long time to achieve a significant effect. Further work will be necessary to identify and mechanistically validate cells and pathways targeted by CASIN in aged mice that are causatively involved in extending lifespan and which are likely epigenetic in nature.

## 2 EXPERIMENTAL PROCEDURES

### 2.1 Mice

All mice included in the study were females C57BL/6 and were obtained from the internal divisional stock (derived from C57BL/6j mice obtained from both The Jackson Laboratory and NIA/Charles River as well as from C57BL/6JRj mice from Janvier). For the longevity study, 40 mice were randomly selected at 70 weeks of age. Mice were weighed and bled before the beginning of the treatment (day 0) and at days 7 and 35. Mice that failed to recover from blood sampling and mice that died due to laboratory errors were excluded. Mice that needed to be euthanized because they were scored as “weak and about to die” according to our approved animal license protocol for evaluating mouse health status remained part of the dataset. Allocation to control or treated group was done randomly (20 mice each experimental group).

Median lifespan, 95% confidence intervals, and survival analysis were calculated by Prism GraphPad v7.0c. Of note, the median lifespan of our control group matches data obtained for C57BL/6j mice in a large set of lifespan studies of diverse inbred mouse strains (Yuan et al., 2009). Young C57BL/6 mice were 10‐week‐old and were hosted in the same room and setting of the old mice. Animals were housed in groups of up to 4 animals per cage with bedding and paper nesting material with a temperature range of 22 ± 1°C and a relative humidity of 55 ± 10%. Animals had access to food (V1124‐3, ssniff^®^) and water ad libitum. Animals were kept at a day/night rhythm of 12/12 h, a temperature range of 22 ± 1°C, a relative humidity of 55 ± 10%, an air exchange rate of 15 times throughout the entire experiment. All mouse experiments were approved by the Regierungspraesidium Tuebingen, Baden‐Württemberg, and the IACUC at CCHMC, USA (protocol numbers: 35/9185.81‐3/1363 and 1472 and IACUC2017‐0096).

### 2.2 CASIN solution and IP treatment

CASIN was prepared fresh before injection by dissolving the drug directly in beta‐cyclodextrin solution (Sigma #H5784). IP injections were done in the morning every 24 h for 4 consecutive days during week 75 of age of the mice. Control mice were injected with equal volume of the solvent. Serum for the cytokine array was prepared from the day 7 bleeding point. Serum for mass spectrometry analysis of CASIN levels was prepared by bleeding an equal (for sex, strain, origin, and age) cohort of mice 3, 24, and 48 h after the end of the treatment. The same stock of CASIN was used for the whole study. CASIN was provided as lyophilized powder.

### 2.3 Flow cytometry of PB

PB cell immunostaining was performed according to standard procedures, and samples were analyzed on a LSR II flow cytometer (BD Biosciences). For PB lineage analysis, the antibodies used were all from eBioscience: anti‐CD3ε (clone 145‐2C11), anti‐B220 (clone RA3‐6B2), anti‐Mac‐1 (clone M1/70), and anti‐Gr‐1 (clone RC57BL/6‐8C5). Lineage FACS analysis data are plotted as the percentage of B220^+^, CD3^+^, and Myeloid (Gr‐1^+^, Mac‐1^+^, and Gr‐1^+^ Mac‐1^+^) cells among total white blood cells. WBC, RBC, Ly, NE, Mo counts were generated by using the hemocytometer Hemavet 950, Drew Scientific Inc, (FL 33014), USA.

### 2.4 Liquid Chromatography–Mass Spectrometry (LC‐MS/MS) measurements of CASIN pharmacokinetics in mouse serum

A LC/MS/MS ion chromatography protocol was employed to measure serum CASIN concentration ranging 0.1–10 μM by comparing serum CASIN from i.p. injected mice at varying times with the standard curve derived by in vitro spiking mouse serum with defined doses of CASIN. An autosampler maximum recovery vials with caps (Waters), an UPLC‐MS system (Waters Quattro Premier XE mass spectrometry with ACQUITY UPLC system), an ACQUITY BEH C18 UPLC column (2.1 × 75 mm, 1.7 µm) (Waters), and an ACQUITY BEH C18 UPLC guard column (Waters) were used. For LC gradient, solvent A contains acetonitrile/water (5/95) with 2 mM ammonium acetate and solvent B contains acetonitrile /water (90/10). The gradient mobile phase switches from 60% solvent A to 57% solvent A over 2 min, to 50% solvent A at 2.1 min, to 46% solvent A over 9.9 min, and then, after changing to 30% at 12.1 min, to 1% solvent A over 5.9 min, then to 60% solvent A at 18.1 min, and this was held for 2 min. Column temperature was kept at 25°C. The capillary voltage is 3 KV for ES‐ mode, cone voltage is 45 V, collision 30 V, desolvation temperature, 350°C; desolvation gas flow is 600 L/h; source temperature is 120°C; and MRM transition m/z is 305.1‐>244.

### 2.5 Cytokine array. Luminex Method

Cytokine concentrations in the sample supernatants were determined by using MilliplexTM Multiplex kits (MilliporeSigma, Darmstadt, Germany) according to the manufacturer's protocol. Briefly, in a 96‐well black plate, 25 µl sample in duplicate was incubated with 25 µl antibody‐coated beads overnight at 4C on a plate shaker. Plates were then washed 2 times using the BioTek 405 TS (BioTek, Winooski, VT), and 25 µl of secondary antibody was added and incubated at room temperature for 1 h while shaking. Finally, 25 µl of streptavidin‐RPE was added directly to the secondary antibody and incubated for 30 min at room temperature with shaking. Plates were then washed 2 more times, and 150 µl of sheath fluid was added. Plates were shaken for 5 min and then read using luminex technology on the Milliplex Analyzer (MilliporeSigma, Darmstadt, Germany). Concentrations were calculated from standard curves using recombinant proteins and expressed in pg/ml.

### 2.6 Cdc42‐GTPase effector domain pull‐down assays

Relative levels of GTP‐bound Cdc42 were determined by an effector pull‐down assay. Briefly, lineage‐depleted BM cells (10^6^) were lysed in a Mg^2+^ lysis/wash buffer (Upstate Cell Signaling Solutions) containing 10% glycerol, 25 mM sodium fluoride, 1 mM sodium orthovanadate, and a protease inhibitor cocktail (Roche Diagnostics). Samples were incubated with PAK‐1 binding domain/agarose beads, and bound (activated) as well as unbound (non‐activated) Cdc42 fractions were probed by immunoblotting with an anti‐Cdc42 antibody (Millipore, rabbit polyclonal). Activated protein was normalized to total protein and/or β‐actin (Sigma), and the relative amount was quantified by densitometry.

### 2.7 Analysis of the epigenetic aging signature

CASIN (Xcessbio #M60040) was prepared freshly before injection. To this, it was dissolved in DMSO to a concentration of 100 mM and diluted in cyclodextrin solution (Sigma #H5784). IP injections of 25 mg/kg were done on 4 consecutive days in the morning starting at an age of 77–86 weeks. Control mice were injected with equal volume of the solvents. Mice were sacrificed 8–9 weeks after treatment, and blood was collected by heart puncture. The analysis of DNA methylation levels was analyzed at three age‐associated CG dinucleotides (CpGs) as described previously (Brown et al., 2020; Han et al., 2018). Briefly, genomic DNA was isolated from blood samples, bisulfite converted, and DNA methylation was analyzed within the three genes (*Prima1*, *Hsf4*, and *Kcns1*) by pyrosequencing. The DNA methylation results at these sites were integrated into a multivariable model for epigenetic age predictions in B6 mice, which clearly correlate with the chronological age (Han et al., 2018). Chronological and biological age of CASIN‐treated and control mice was compared using an unpaired *t* test. Since the data were not normally distributed, all data were potentiated to obtain normal distribution before the statistical analysis was performed.

## CONFLICT OF INTEREST

W.W. is cofounder of Cygenia GmbH (www.cygenia.com), which can provide service for epigenetic age predictions to other scientists. Y.H. contributes to this company, too. All other authors do not have a conflict of interest to declare.

## AUTHOR CONTRIBUTIONS

M.C.F. designed the experiments and analyzed the data. H.L. designed experiments and analyzed the data. V.S., K.N., G.M., and K.S. performed mouse experiments. A.V. was responsible for serum preparation for the cytokine array. K.N. supported the cytokine array analyses. A.R., X.Z., and K.S. performed mass spectrometry quantification of CASIN in serum. W.W., M.G., and Y.H. performed methylation analyses. M.M. performed statistical analyses. Y.Z. supervised experiments and interpreted data. H.G. and M.C.F. supervised the experiments, interpreted data, and wrote the manuscript.

## Supporting information

Figures S1‐S2

## Data Availability

The data that support the findings of this study are openly available in Dryad digital repository at https://datadryad.org/review?doi=doi:10.5061/dryad.c4798q5.
