# Supplementary figures and images for: Inhibition of Cdc42 activity extends lifespan and decreases circulating inflammatory cytokines in aged female C57BL/6 mice
Source: Aging Cell. 2020 Aug 4;19(9):e13208. doi: 10.1111/acel.13208 (PMC7511875; doi:10.1111/acel.13208)

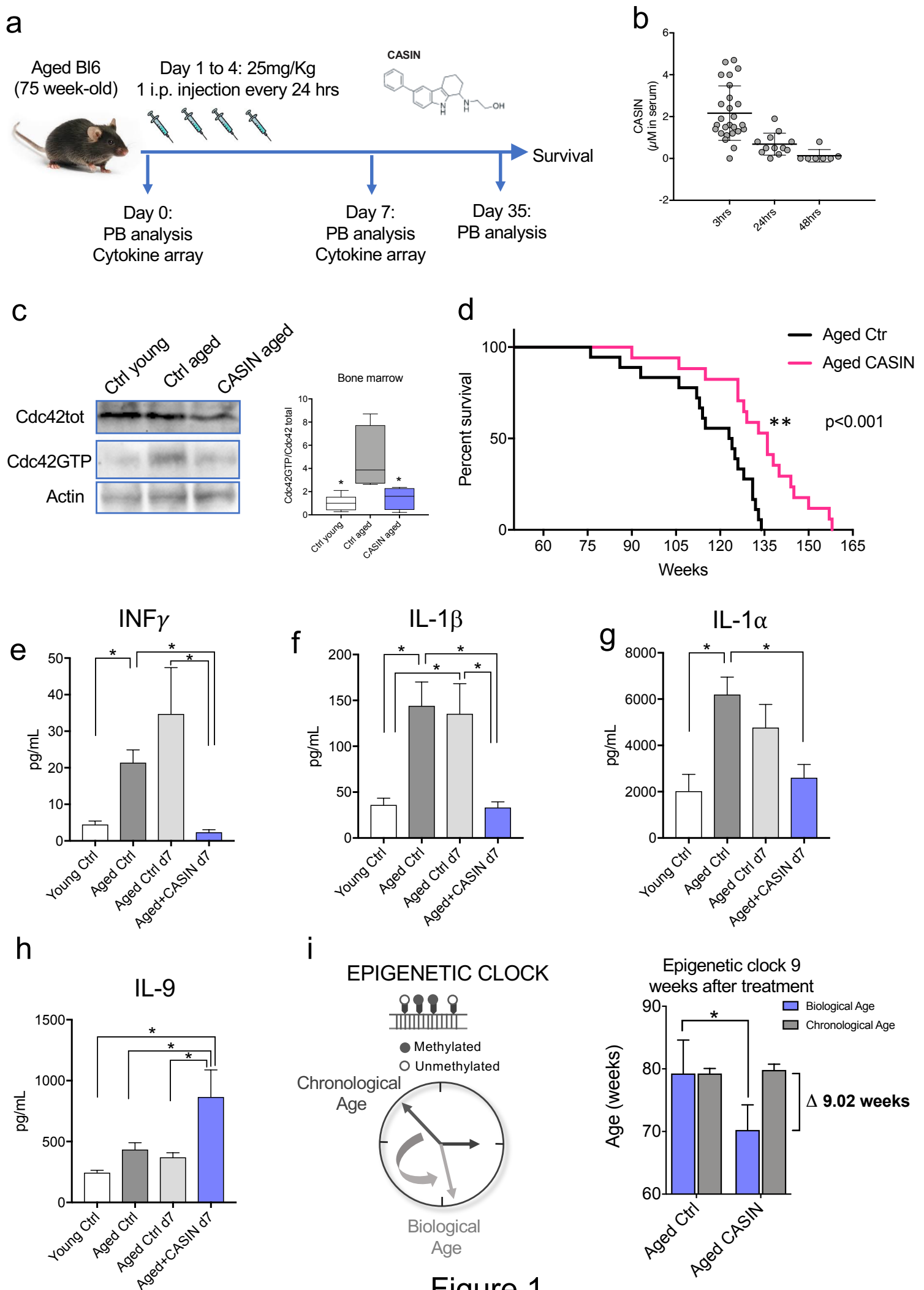

Figure 1

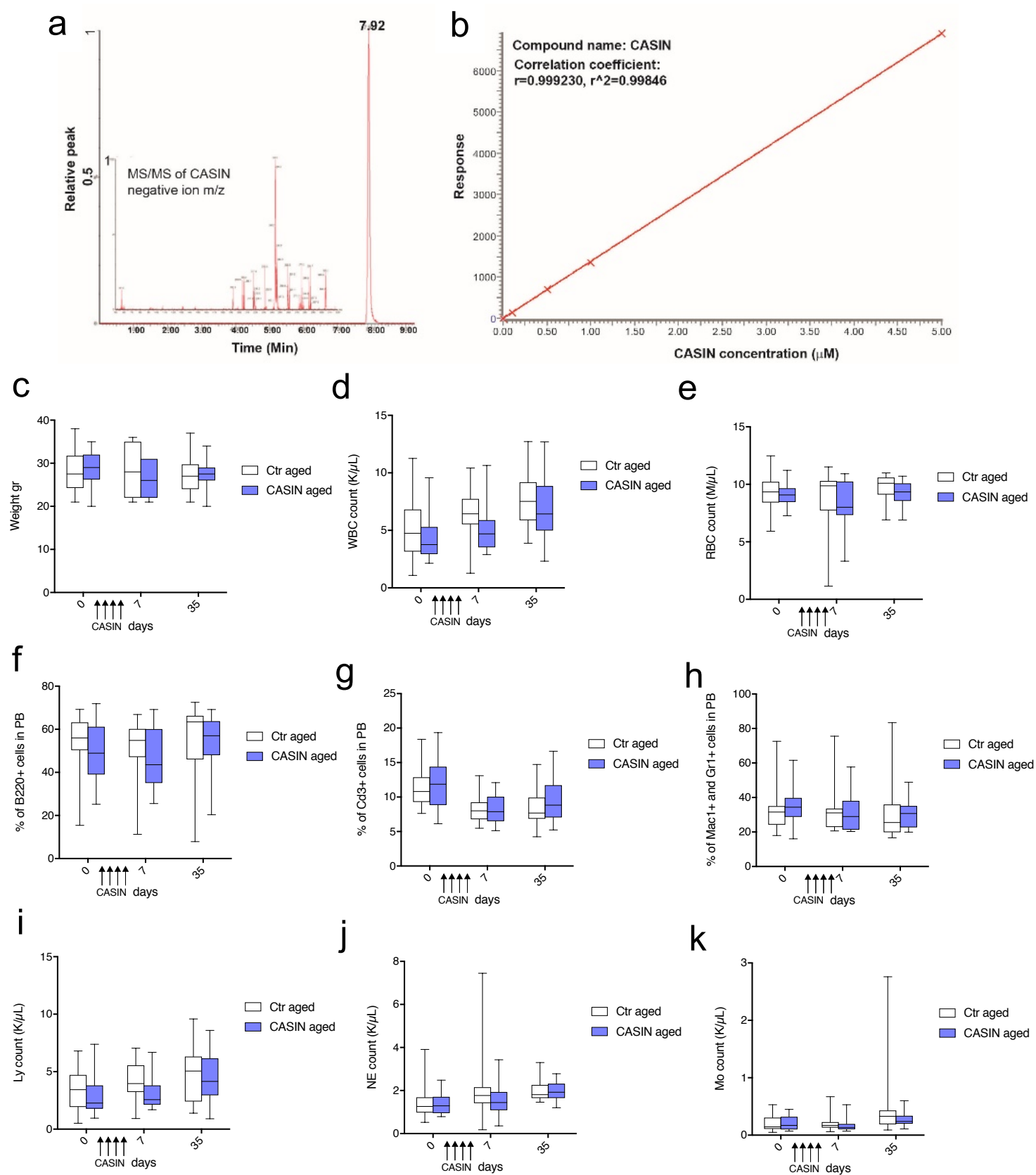

Supplementary Figure 1

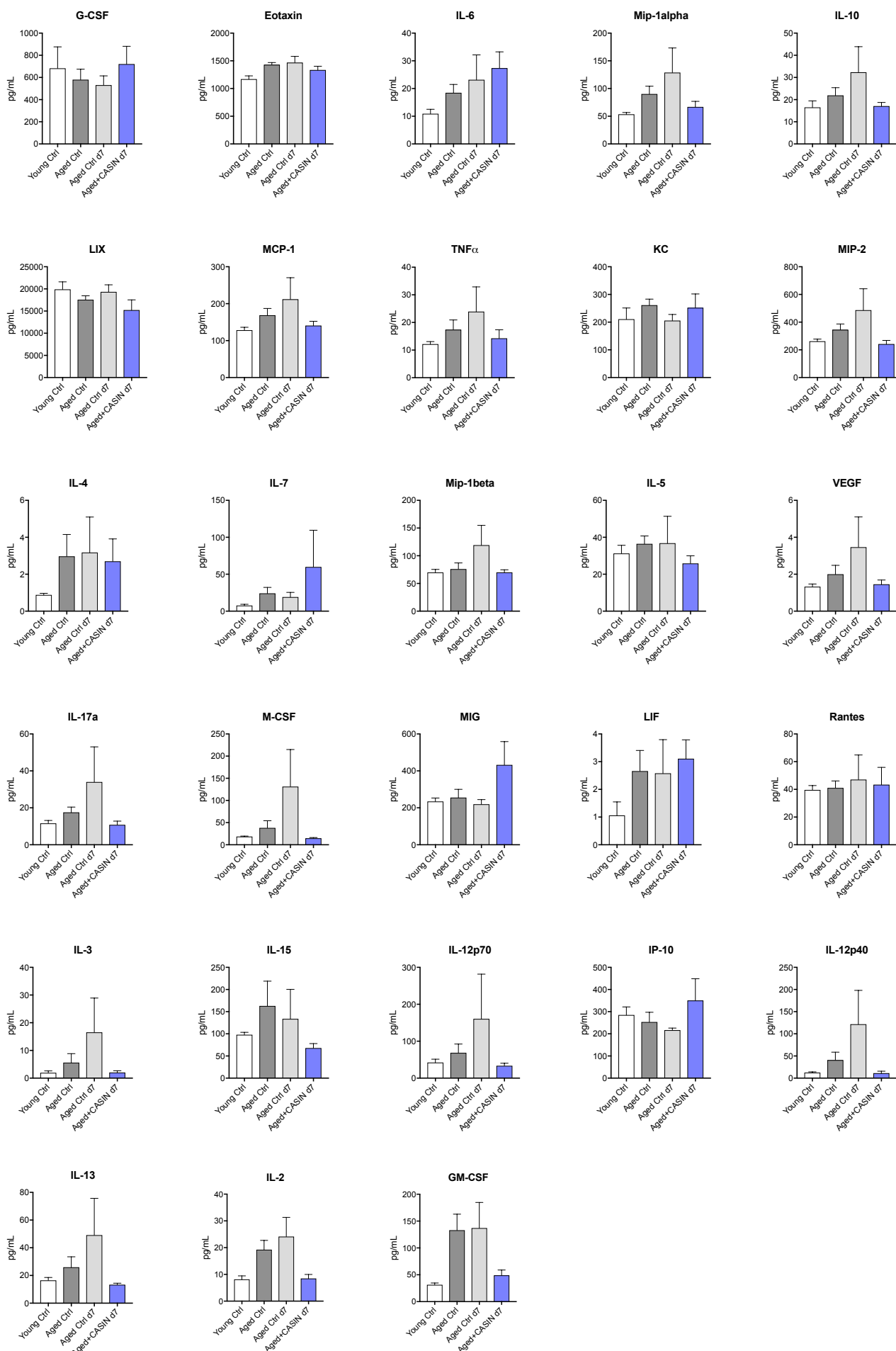

Supplementary Figure 2

Supplement: Supplementary file 1 — Figures S1‐S2 [file ACEL-19-e13208-s001.pdf]
